# Broad recruitment of mGBP family members to *Chlamydia trachomatis* inclusions

**DOI:** 10.1371/journal.pone.0185273

**Published:** 2017-09-25

**Authors:** Valesca Lindenberg, Katja Mölleken, Elisabeth Kravets, Sonja Stallmann, Johannes H. Hegemann, Daniel Degrandi, Klaus Pfeffer

**Affiliations:** 1 Institute of Medical Microbiology and Hospital Hygiene, Heinrich-Heine-University Düsseldorf, Universitätsstrasse 1, Düsseldorf, Germany; 2 Institute for Functional Microbial Genomics, Heinrich-Heine-University Düsseldorf, Universitätsstrasse 1, Düsseldorf, Germany; University of California, San Francisco, UNITED STATES

## Abstract

Chlamydia, the most common sexually transmitted pathogen, is an exquisitely adapted Gram-negative obligate intracellular bacterium. Intracellular *Chlamydia trachomatis* replicate in a specialized vacuole, termed inclusion, which shields the bacterium from antimicrobial immunity of the host cells and acts as a signalling interface. Previously it was shown that members of the interferon induced guanylate binding protein (mGBP) family, in particular murine GBP1 and mGBP2, were found to accumulate at the bacterial inclusions, similar to previously published recruitment of GBPs to the parasitophorous vacuole of *Toxoplasma gondii*. Here, we provide a wide comparison of mGBPs roles within the host cell in the context of Chlamydia and Toxoplasma infection. By confocal microscopy on fixed and living infected cells we show localization of mGBP3, mGBP6, mGBP7, mGBP9, and mGBP10, in addition to mGBP1 and mGBP2, at chlamydia inclusions. In time lapse videos using GFP expressing Chlamydia we show rapid and transient dynamics of mGBP9 accumulation onto chlamydia inclusions. Taken together this study reveals a broad activation of mGBP recruitment towards *Chlamydia trachomatis* inclusions after infection and provides evidence for time limited action of mGBP9 at the chlamydia inclusion.

## Introduction

The host response against intracellular pathogens is orchestrated by Interferon γ (IFNγ), an important pro-inflammatory cytokine which induces a wide range of genes, amongst which genes of the Guanylate Binding Proteins (GBPs) are very abundantly expressed [1–3]. GBPs are a family of GTPases of approximately 67 kDa which localize at specialized compartments in the cytosol of mammalian cells [4–9]. Through GTP hydrolysis via GDP to GMP the GBPs have the ability to form multimers in vesicle like structures (VLS) and accumulate into supramolecular complexes around pathogen containing vacuoles [10–15]. It was shown by FRET-based interaction studies in living cells that murine GBP2 (mGBP2) multimerizes predominately with mGBP1 and mGBP3, but not with mGBP5 or mGBP6 [11]. Various immune functions of mGBPs in various effector pathways have been described, for example assembling oxidative, autophagic or inflammasome complexes [13, 16–19]. The effector functions of GBPs have been thoroughly studied in *Toxoplasma gondii* (*T*. *gondii*) infection [4, 11, 12, 20–22].

*T*. *gondii* is an apicomplexan parasite which actively invades the host cell, shaping a parasitophorous vacuole (PV) and replicating within, thus being shielded from part of the cell autonomous immunity [23]. Nonetheless, if mice are deficient for a cluster of GBPs on chromosome 3, as well as for mGBP1 or mGBP2 individually they are more susceptible to *T*. *gondii* infection [20–22]. *In vitro*, mGBPs were shown to accumulate onto PVs, disrupt or permeabilize the PV membrane and to target the plasma membrane of parasites [24]. Interestingly, comparable observations were made *in vitro* for the bacterial pathogen *Chlamydia trachomatis* (*C*. *trachomatis*) [24].

*C*. *trachomatis* is a Gram-negative bacterium with a biphasic lifecycle [25]. The small infectious particles termed elementary bodies (EB) invade epithelial cells via an endocytosis-like mechanism, thus forming an intracellular compartment termed ‘inclusion’ [25]. Therein the EBs differentiate into metabolically active reticulate bodies (RBs) which extensively alter the inclusion membrane and multiply by binary fission [26–28]. By subverting cellular protein trafficking the inclusions increase in size until they rupture [29]. Despite long co-evolution between hosts and chlamydia potent immune pathways, such as inflammasome signalling, are capable of combatting the pathogen [24]. Towards chlamydia inclusions, an accumulation of mGBPs similar to recruitment of various mGBPs to toxoplasma containing vacuoles was shown for mGBP1 and mGBP2 [30, 31]. Accumulation of mGBP2 on chlamydia inclusions has been shown to be dependent on autophagy related proteins, and associated with polyubiquitination around the pathogen compartment [32, 33].

Only limited information about mGBPs exists in this rapidly advancing field, studying immunity to intracellular pathogens. Therefore we extensively explored the mGBP family for accumulation to both *C*. *trachomatis* as well as *T*. *gondii*. This extensive analysis revealed that although the majority of mGBPs can localize towards intracellular pathogens, an ample role exists for mGBP6 in apicomplexan and mGBP9 in bacterial infection. For the first time live cell time lapse analysis displays the dynamics of mGBPs accumulation onto chlamydia inclusions.

## Materials and methods

### Host cell culture, bacterial and protozoan strains and infections

NIH 3T3 murine fibroblasts (ECACC 93061524) and 293FT (Thermo Scientific P/N 51–0035) were cultured in Dulbecco’s modified Eagle’s medium (DMEM, Gibco) supplemented with 10% heat-inactivated fetal bovine serum (FBS; Pan Biotech) and 0.05 mM β-mercaptoethanol (Gibco). In order to visualize mGBPs a lentiviral genetic transfer protocol was utilized as previously described [20]. Briefly, 293FT cells were transiently co-transfected with expression vector pWPXL [11] containing an individual mGBP N-terminally fused with mCherry- or GFP, a lentiviral envelope vector pLP/VSVG (Invitrogen, Germany) and the packaging vector psPAX2 (D. Trono Laboratory, Lausanne, Switzerland) at equimolar ratios, using jetPRIME (Polyplus) according to the manufacturers’ instructions. After 48 hrs lentivirus was harvested, mixed with 40 μg/ml Polybrene (Merck Millipore) and used to transduce fibroblasts of which GFP or mCherry positive cells were sorted using flow cytometry, and cultivated further.

*C*. *trachomatis* L2/434/Bu (ATCC VR-902B) and *C*. *trachomatis* L2/434/Bu transformed with pGFP::SW2 [34] were propagated in HEp-2 cells (ATCC CCL-23) cultured in DMEM medium supplemented with 10% fetal calf serum (FCS), MEM vitamins, non-essential amino acids (Thermo Scientific). Chlamydia elementary bodies (EBs) were purified using a 30% gastrographin solution (Bayer) and stored in SPG buffer (220 mM sucrose, 3,8mM KH2PO4, 10,8 mM Na2HPO4, 4,9 mM L-glutamine). Infections with *C*. *trachomatis* were performed with NIH 3T3 murine fibroblasts constitutively expressing mGBPs fused to GFP or mCherry seeded on glass coverslips (Ø 1cm) at a nominal multiplicity of infection (MOI) of 3 with centrifugation for 1 hour at 2900 rpm (Rotanda, Hettich). After 3 hours incubation cells were stimulated by adding fresh media containing IFNγ (R&D Systems) to reach a final concentration of 100 U/ml and incubated for 11 more hours at 37°C and 8% CO_2._

*T*. *gondii* tachyzoites of the type II strain ME49 were maintained by serial passage as described [4]. Cells were stimulated overnight with 100 U/ml IFNγ (R&D Systems) and infected with *T*. *gondii* tachyzoites at a nominal MOI of 50.

### Immunofluorescence analyses

Cells were washed with phosphate buffered saline (PBS) (Gibco), pH 7.4 and fixed with 4% paraformaldehyde (PFA; Sigma-Aldrich) for 10 minutes and subsequently washed. Chlamydia and Toxoplasma infected samples were permeabilized with methanol (VWR Chemicals) for 5 minutes and washed or 0.02% saponin (Calbiochem-Merck) for 15 minutes, respectively. Blocking was performed with 2% Goat serum (DaKo) in PBS or 0,002% saponin, respectively. Subsequently cells were stained with a primary antibody against *T*. *gondii* Sag1 (Abcam) at 1:700: or *C*. *trachomatis* Momp (BIOD166, Santa Cruz) at 1:100, rabbit polyclonal antibody against CT868 (S1 Fig) at 1:25, or rabbit polyclonal antibody against IncA [35] at 1:500, followed by washing and Alexa Fluor 488- or Cy3-conjugated secondary antibodies (Jackson immunoResearch) at 1:200. Nucleic and pathogen DNA were stained with 4’,6-diamidino-2-phenylindole (DAPI; Invitrogen Life Technologies) at 1:1000. Stained cells were washed and mounted on microscope slides with FluoromountG (Southern Biotechnology Associates) and allowed to settle overnight. All cells were imaged using an LSM780 confocal microscope (Zeiss) equipped with live cell imaging technology. Because of the highly different staining intensities of host cell and bacterial DNA, the DAPI channel was configured to allow imaging of chlamydial DNA, leading to saturation of the host cell nucleus.

Recruitment of proteins towards pathogens was quantified in three independent experiments. For each mGBP expressing cell line, around 200 randomly chosen inclusions or PVs were manually counted for co-localization with each individual mGBP. Differential interference contrast (DIC) was used to identify and exclude extracellular *T*. *gondii* tachyzoites.

### Live-cell imaging

For Live cell microscopy, cells were seeded on imaging dishes CG (MoBiTec), cultured in Phenol-free or FluoroBrite cell culture media (Gibco) and infected with *C*. *trachomatis* L2/434/Bu transformed with pGFP::SW2 at an MOI of 30. 3hpi cells were stimulated with IFNγ as described above. Imaging started immediately after IFNγ stimulation at 3hpi at 37°C with 8% CO_2_ and humidity saturated air. Image analysis was performed using ZEN (Zeiss) and Imaris (Bitplane) software.

### Statistical analysis

Results are represented as means +SEM as indicated. The unpaired two-tailed Student’s t-test was used to determine the statistical significance of the experimental data compared to the mGBP2 isoprenylation mutant C586S, which lost the ability to interact with membranes [20]. p≤0.05 was considered significant.

## Results

### Colocalization of mGBPs at chlamydia inclusions

**Fig 1:**
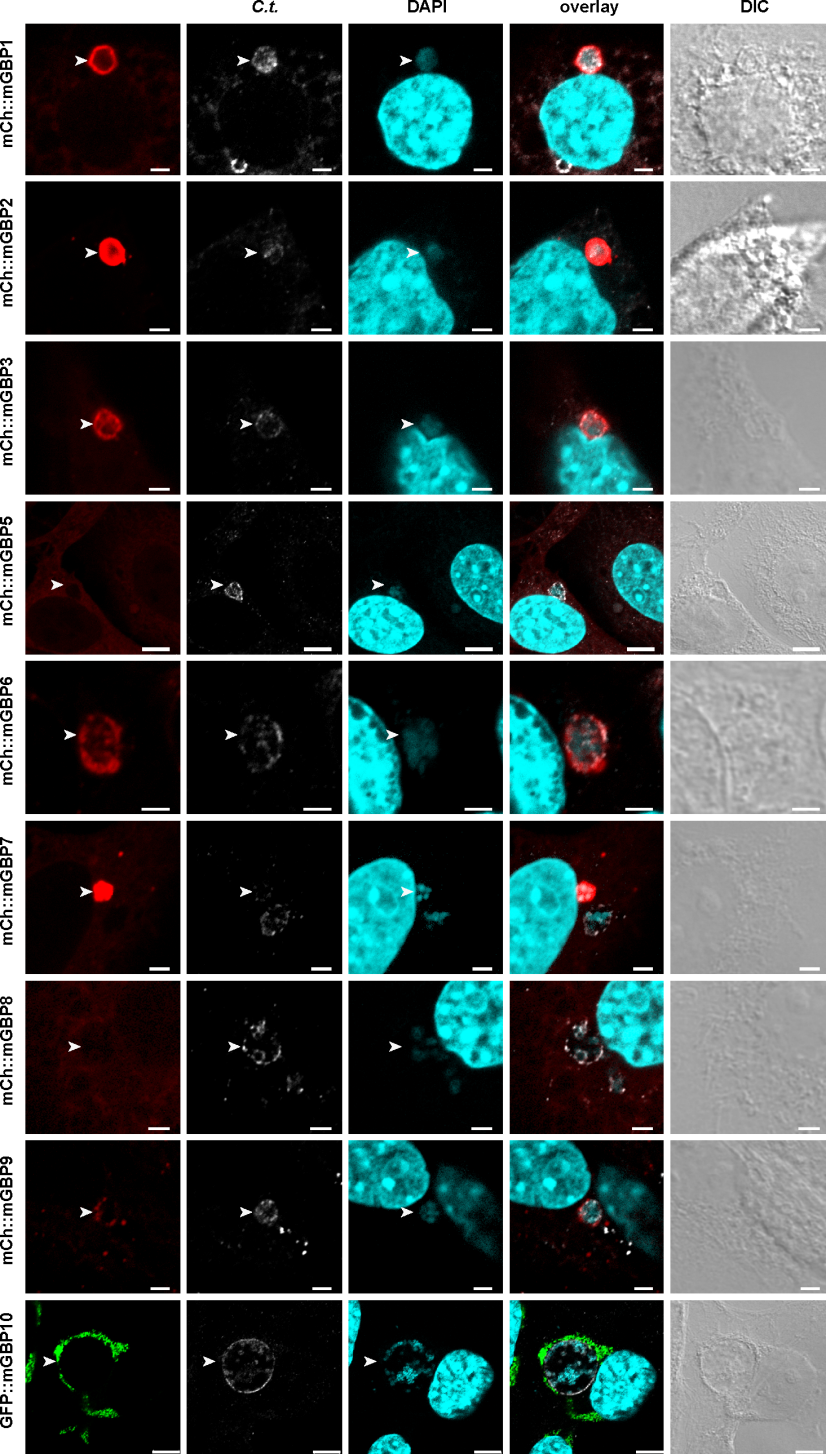
Colocalization of mGBPs with *C*. *trachomatis* inclusions. NIH 3T3 fibroblasts which stably express individual N-terminal fluorescent protein-fused mGBPs were infected with *C*. *trachomatis* (*C*.*t*.) and treated with IFNγ at 3 hpi. Cells were fixed at 14 hpi, stained for chlamydia inclusion membrane marker CT868 together with DAPI. Cells were analysed by confocal microscopy and representative images are shown, bar = 2 μm.

Previously, mGBP1 and mGBP2 were found to locate to an intracellular pathogen compartment commonly referred to as ‘inclusion’ in *C*. *trachomatis* infected cells [30]. Based on homologies between mGBP sequences [4] and the ability of mGBPs to form multimers [11], we hypothesized that additional mGBPs might be involved in anti-bacterial resistance to *C*. *trachomatis*. To extensively analyse mGBPs, each fluorescent protein-tagged mGBP was stably transduced into the NIH 3T3 fibroblast cell line. These cells were infected with *C*. *trachomatis*, stimulated with interferon gamma (IFNγ), fixed at 14 hours post infection (hpi) and subsequently stained. Without IFNγ treatment no accumulation of mGBPs at inclusions could be observed. In order to discriminate the host-pathogen interface, the novel inclusion membrane marker CT868 (also known as ChlaDUB1 and Cdu1) (S1 Fig) [36], was stained and colocalization with fluorescent protein-tagged mGBPs was assessed. Via confocal laser scanning microscopic (LSM) analysis it could be shown that besides mGBP1 and mGBP2, also mGBP3, mGBP6, mGBP7, mGBP9, and mGBP10 are found at the inclusion-membrane (Fig 1). High-resolution Z-stack and colocalization analysis performed exemplarily for mGBP2 confirms colocalization of mGBP2 with CT868 (S2 Fig). Among accumulation events, a distinct morphology was observed for mGBP7, which was characterized by intense and tight accumulation onto the inclusion membrane. In the case of mGBP5, a more diffuse localization around the inclusion was observed. For mGBP8 virtually no colocalization with inclusions could be found. When mGBPs co-localize at an inclusion, it was observed that α-CT868 stained to a lesser extend compared to inclusions without colocalization (See Fig 1 panel mCh::mGBP7). To compensate for this loss of chlamydia inclusion membrane signal all inclusions were also assessed for the presence of bacterial DNA defined by DAPI staining in the cytoplasm of infected cells. Taken together, the mGBPs with the ability to localize towards chlamydia can be extended from mGBP1 and mGBP2, to the additional family members mGBP3, mGBP6, mGBP7, mGBP9 and mGBP10.

### Colocalization of mGBPs with toxoplasma

**Fig 2:**
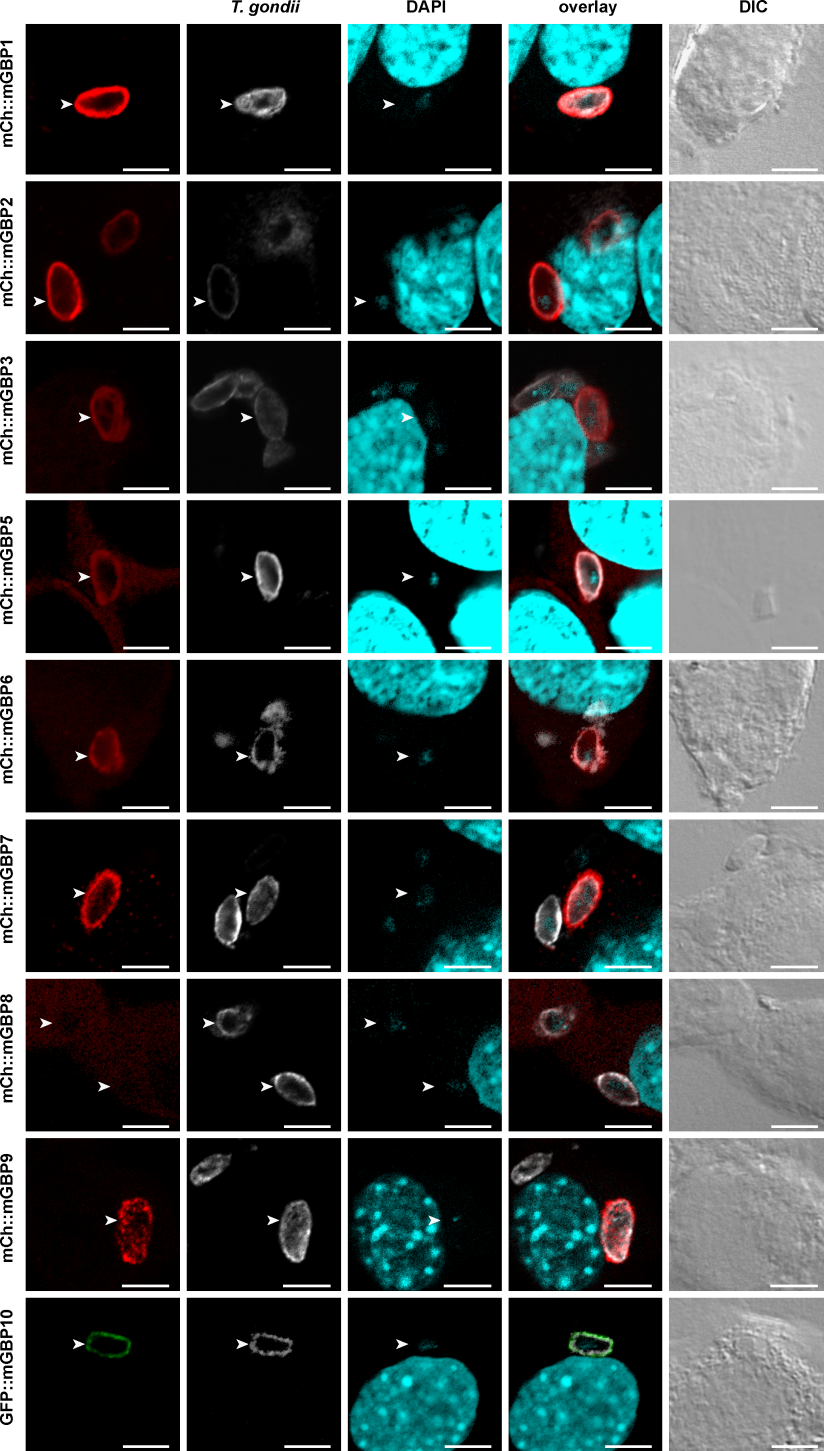
Colocalization of mGBPs with intracellular *T*. *gondii*. NIH 3T3 fibroblasts which stably express individual fluorescent protein-fused mGBPs were treated for 16 hours with IFNγ. Cells were infected with *T*. *gondii* for 2 h, stained for SAG1 and nucleic acids with DAPI. Glass slides were analysed by confocal microscopy. Representative images, bar = 5 μm.

To consistently compare the role of individual mGBPs in infections with bacterial and apicomplexan pathogens, the mGBP-expressing NIH3T3 cells described above were infected with *T*. *gondii* strain ME49. Infections were performed for 2 hours in cells pre-stimulated for 16 hours with IFNγ. In these stably transduced cell lines, constitutively expressed fluorescent protein-tagged mGBPs were observed at the intracellular *T*. *gondii* PV (Fig 2). Previous observations that mGBP1, mGBP2, mGBP3, mGBP5, mGBP6, mGBP7 and mGBP9 recruit towards PVs in MEFs and RAW264.7 cells [4, 11] could be confirmed via this alternative experimental strategy. The morphology of mGBP5 accumulation distinguishes itself by localising around the PV with a dispersed morphology, comparable to its morphology when localising around chlamydia inclusions. In addition, previous findings [4] were now extended to mGBP8 and mGBP10, which were found to hardly recruit (mGBP8) and to recruit (mGBP10) towards PV. Therewith a comprehensive analysis of mGBPs recruitment to *T*. *gondii* PVs shows that many, but not all protein family members play a role in cellular resistance against apicomplexan pathogens.

### Quantification of mGBP colocalization reveals different frequencies of recruitment

**Fig 3:**
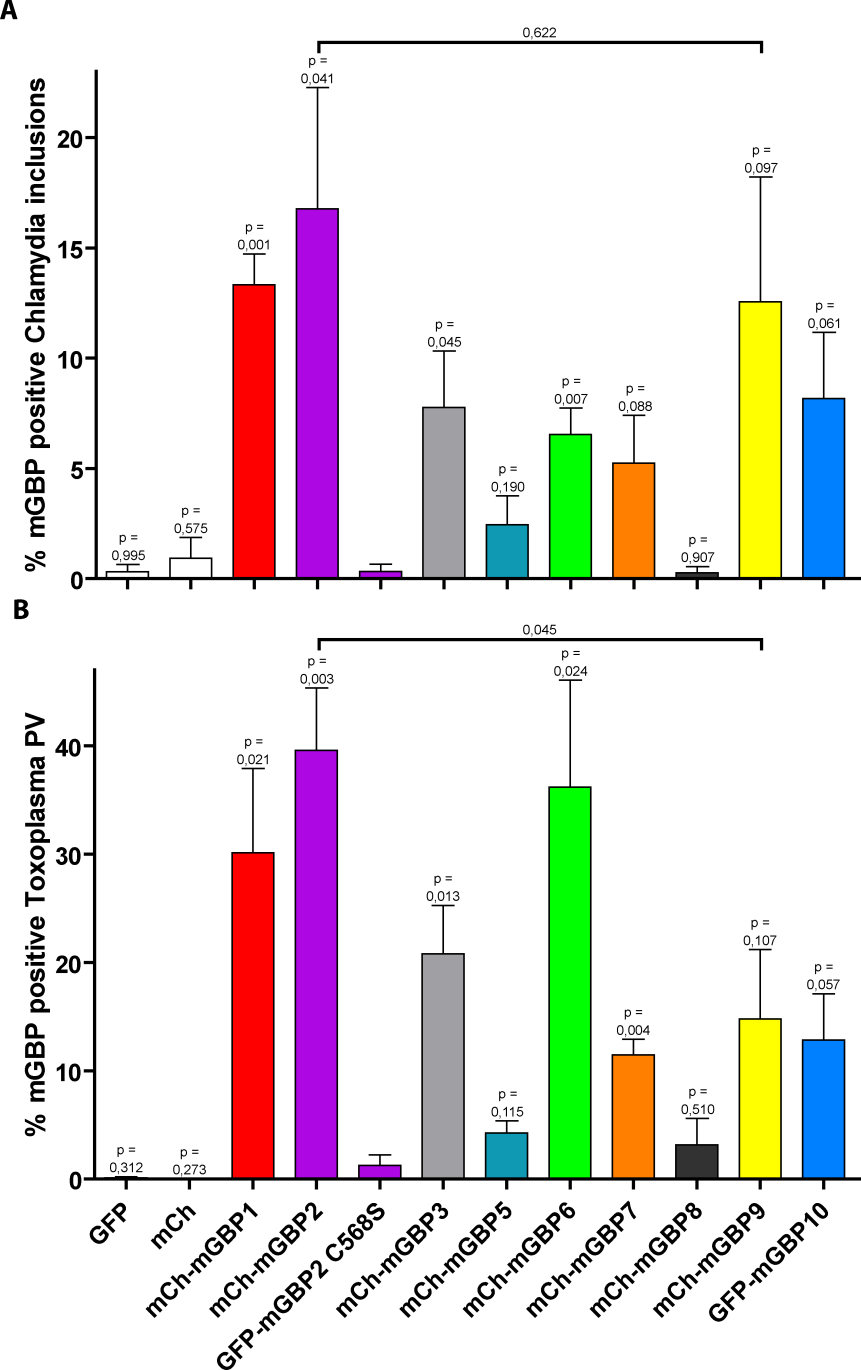
Quantification of mGBP recruitment towards intracellular pathogens. (A) NIH 3T3 fibroblasts which stably express individual fluorescent protein-fused mGBPs were infected with *C*. *trachomatis* and treated with IFNγ at 3 hpi. Cells were fixed at 14 hpi and stained for chlamydia inclusion membrane marker CT868 and with DAPI. Via confocal microscopy, for each mGBP expressing cell line, around 200 randomly chosen inclusions were manually counted for co-localization with each individual mGBP. (B) NIH 3T3 fibroblasts which stably over-express individual fluorescent protein-fused mGBPs were treated for 16 hours with IFNγ. Cells were infected with *T*. *gondii* for 2 h, stained for SAG1 and nucleic acids with DAPI. Using confocal microscopy around 200 cells were assessed for the presence of intracellular parasites via DIC, and subsequently for co-localization with individual mGBPs. (A and B) Data of three independent experiments are depicted as mean percentages + SEM.

To assess the relative contribution of individual mGBPs at the pathogen containing intracellular compartments we proceeded to quantify the recruitment events in both bacterial and apicomplexan infection models (Fig 3). For *C*. *trachomatis* co-localization of mGBP1 and mGBP 2 with inclusions closely resembled rates described previously [31, 33, 37]. The mGBP2 C586S mutant showed virtually no colocalization (S3 Fig), proving that isoprenylation is essential for localization towards chlamydia inclusions, as it has been described for recruitment to toxoplasma PVs [11, 20]. By relating each mGBP to mGBP2 C586S we could separate the GBPs shown to localize to inclusions in Fig 1 into different groups based on their distinct frequency of colocalization. Thus, mGBP1 and mGBP2, as well as mGBP3 and mGBP6 colocalized to a significant degree with inclusions. Due to higher variation mGBP7, mGBP9 and mGBP10 showed a trend to localize considerably often towards inclusions. Remarkably, in a few experiments mGBP9 colocalized with chlamydia more frequently than mGBP2. The third group consists of mGBP5 and mGBP8, which hardly localized towards chlamydia inclusions.

Quantifying the recruitment towards *T*. *gondii* revealed a remarkable similarity to the recruitment profile towards chlamydia inclusions, which is underlined by a high correlation (Kendall’s tau coefficient = 0.6667; p-value = 0.0018). However, a few differences were observed. mGBP1, mGBP2, mGBP3, mGBP6 and mGBP7 colocalized with high frequencies to PVs. Interestingly, in this infection the highest recruiter aside form mGBP2 was mGBP6. Considerably often mGBP9 and mGBP10 recruited to PVs, whereas mGBP5 and mGBP8 hardly recruited towards *T*. *gondii*. Overall PV recruitment percentages compared to previous publications [11, 12, 20, 37]. Remarkable is mGBP9, since it showed a comparable frequency of colocalization as mGBP2 in chlamydia infection, whereas in toxoplasma infection it recruited significantly less often than mGBP2. This quantification suggests that not all mGBPs contribute equally towards cell autonomous immunity.

### mGBP9 encloses chlamydia inclusions in living cells

**Fig 4:**
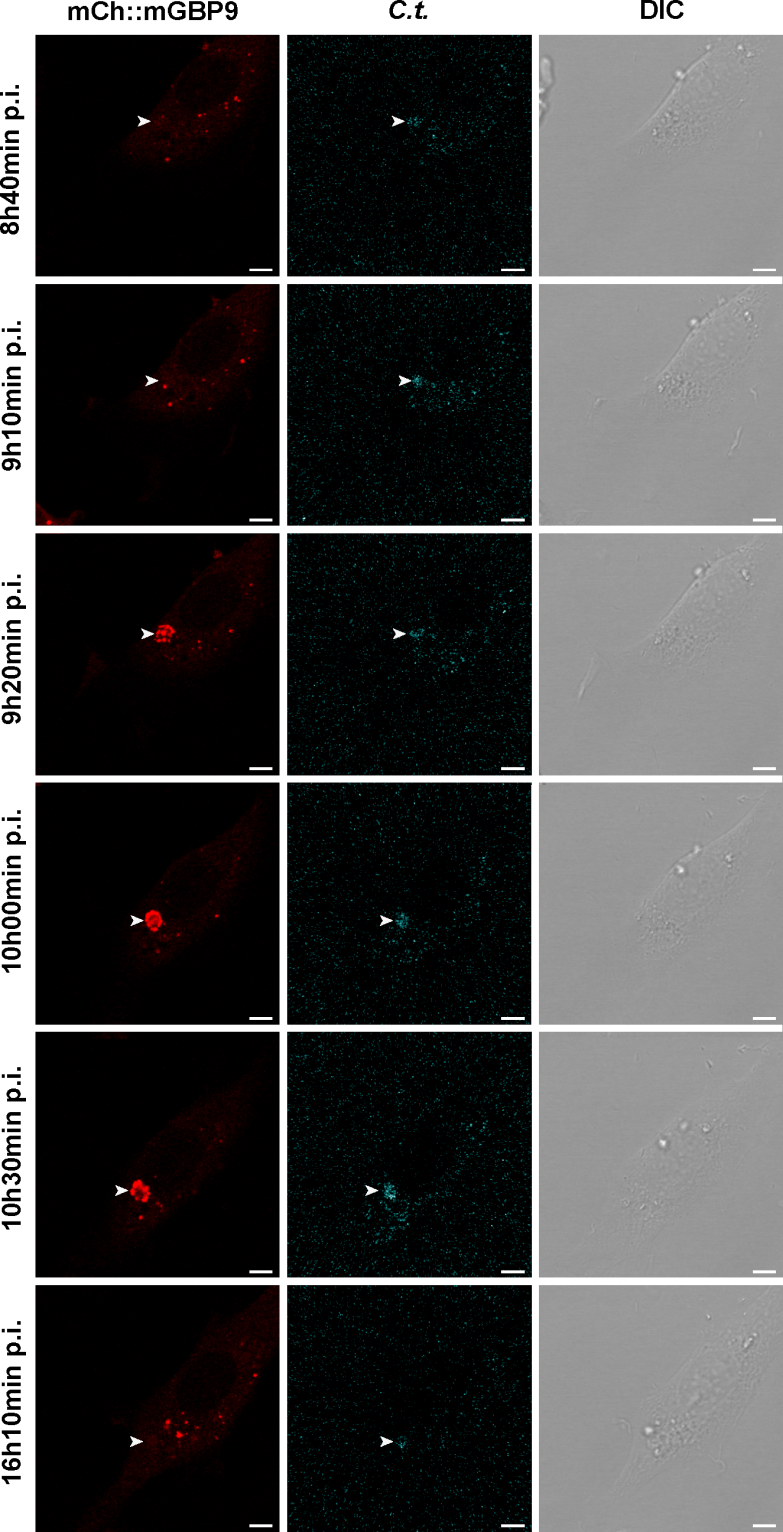
Dynamics of mGBP9 recruitment around the chlamydia inclusion. NIH 3T3 fibroblasts which stably express mCherry-fused mGBP9 were infected with *C*. *trachomatis* SW2::GFP (*C*.*t*.) [34] and stimulated with IFNγ at 3 hpi. The cells were incubated at 37°C with 8% CO_2_ and humidity saturated air while recording confocal LSM images every 10 minutes.

To better understand the dynamics of mGBP recruitment, time lapse recordings of cells during infection were made. *C*. *trachomatis*, containing a plasmid allowing GFP-expression under the SW2 promoter [34], was used to infect cells over-expressing mCherry-tagged mGBP9. We pursued mGBP9 particularly since it appears to have a predominant function in *C*. *trachomatis*, as opposed to *T*. *gondii*, infection (Fig 3A). Imaging started at 3 hpi, the time point where IFNγ was added. In one instance, a larger sized inclusion allowed witnessing mGBP9 accumulation in a cage-like structure at approximately 9h 20min p.i. (Fig 4 and S1 Video, video starts at 6h 40 min p.i.). After approximately 1 hour (10h 30min p.i.) the mGBP9 accumulation reduced in intensity and size until ultimately disappearing (approx 12h p.i.). However, we have also observed mGBP9 remaining at the chlamydial inclusion over the whole observation length (ca. 16h). This analysis shows the exceptional morphology of mGBP9 colocalization with chlamydia in live cell samples, showing for the first time the dynamics of mGBPs in cell autonomous immunity against chlamydia.

## Discussion

*C*. *trachomatis* biovar L2 is a sexually transmittable intracellular bacterium to which cell autonomous immune reactions remain largely undescribed. Although being a human pathogen, *C*. *trachomatis* has been widely used in murine infection studies to unravel antibacterial cell autonomous mechanisms and for studying novel vaccination strategies [38]. In this study, it is shown that not only mGBP1 and mGBP2 [30, 33], but the majority of mGBPs colocalize with chlamydia inclusions. Remarkably, high frequencies of colocalization with chlamydia inclusions were observed for mGBP1, mGBP2, mGBP3, mGBP6, and mGBP9, whereas mGBP7 and mGBP10 also accumulate at the chlamydia inclusion membrane, albeit with lower frequencies. In addition, rare but distinct accumulations of mGBP5 around inclusions were observed with a scattered morphology, whereas mGBP8 does not recruit at all towards the pathogen containing compartments investigated here. This implies that despite high sequence similarities among the mGBPs, individual differences in the response to *C*. *trachomatis* exist. By comparing *C*. *trachomatis* and *T*. *gondii* infection, a specific pattern in recruitment of mGBPs appears, as exemplified by mGBP6 which recruits more frequently to *T*. *gondii* PVs (see Fig 3). Notably, mGBP9 accumulates as often as mGBP2 onto intracellular chlamydial, but not apicomplexan compartments. Previous analyses showed that mGBP2 recruited at the highest frequency compared to other mGBPs to *T*. *gondii* PVs, can destruct the pathogen containing vacuole and impede pathogen viability [4, 11, 12, 30, 31]. This implies that mGBP9 might be an important factor in the clearance of chlamydia.

Results of Kokes & Valdivia in 2015 showed that fixation of chlamydia infected cells with PFA can cause accumulation and translocation of nearby aggregates, organelles, etc. over the chlamydia inclusion membrane [39]. Therefore, in this study, it was investigated whether mGBP9 accumulates onto chlamydia in unfixed, living cells by time lapse recordings. The dynamics revealed that mGBP9 accumulation is a rapid process that takes place approx. 9 hpi (See Fig 4). In addition it showed accumulation can be transient, occurring for a few hours only and can dissolve again as early as 10.5 hpi. Live cell imaging also proves that colocalization with intracellular chlamydia is authentic and provides information which was not available before. Since chlamydia infections occur asynchronously, the colocalization frequency might be vastly underestimated due to quantification at one individual time point. Nevertheless, a large percentage of inclusions is not recognized by mGBPs. It has been proposed that *C*. *trachomatis* can express a factor which interferes with IRG-dependent ubiquitination of inclusions [40], which might also hamper the function of mGBPs. Alternatively, *C*. *trachomatis* might encode additional factors targeting the mGBP defense system. In summary, this study discovered a potential chlamydia-specific role for mGBP9, which must be further explored in the future. Moreover, this study displays the potential of mGBPs encoded on murine chromosome 5, which have up till now not been assigned a role in pathogen defense.

The fact that the majority of GBPs can recruit towards the inclusion membrane of *C*. *trachomatis* is remarkable, since only mGBP1, mGBP2 and mGBP5 contain CaaX-box motives at their C-terminus, allowing isoprenylation and subsequent membrane anchoring. From previous work in the Toxoplasma infection model it is known that mGBP2 recruitment depends on the residue C586, since mutation of this amino acid results in incapacitation of the isoprenylation site and consequently a lack of recruitment to the *T*. *gondii* PV membrane [11, 20]. Here, we found that colocalization with another pathogen, *C*. *trachomatis*, is also dependent on the isoprenylation site (see Fig 3). However, whether nucleotide-binding and GTPase activity are required for localization of mGBP2 to chlamydia, as shown for toxoplasma [11, 12, 20], is currently unknown. By utilizing point- and truncation mutants these questions will be addressed in the future.

This study systematically investigated mGBPs and their capacity to recruit on chlamydia inclusions. It was found that in addition to mGBP1 and mBP2, also mGBP3, mGBP6, mGBP7, mGBP9 and mGBP10 can colocalize with *C*. *trachomatis*. For mGBP9, time lapse videos show the rapid and transient accumulation onto chlamydia thus giving insight into the dynamics.

## Supporting information

S1 Fig CT868 localizes at the *C*. *trachomatis* inclusion membrane. Staining: 24 hpi cells infected with *C*. *trachomatis* were fixed with 3% PFA, permeabilized with 0.5% Saponin and stained with antibodies against Momp and IncA in combination with anti-rabbit Alexa594 and anti-mouse Alexa647. Cells were fixed again in 3% PFA, permeabilized with 0.5% Saponin and CT868 was stained by anti-CT868 directly labeled with FITC. DNA was visualized by DAPI, bar = 5 μm.
(TIF)

S2 Fig Z-stack and colocalization analysis of mGBP2 at the *C*.*trachomatis* inclusion. NIH 3T3 fibroblasts which stably express mCh::mGBP2 were infected with *C*. *trachomatis* and treated with IFNγ at 3 hpi. Cells were fixed at 14 hpi, stained for chlamydia inclusion membrane marker CT868 together with DAPI. Cells were analysed by confocal Airyscan microscopy and a z-stack was performed. A representative cell is shown. Colocalization and surface analysis was performed with Imaris. Bar = 1 μm. (A) left panel: maximum intensity projection of mCh::mGBP2 (red), CT868 (green) and DAPI (cyan). Right panel: computed colocalization of mCh::mGBP2 and CT868 is shown (grey). (B) surface analysis of the inclusion shown in A. Left panel: mCh::mGBP2(red) and CT868 (green). Right panel: computed colocalization of mCh::mGBP2 and CT868 (grey).
(TIF)

S3 Fig No colocalization of the mGBP2 isoprenylation mutant, mCherry or GFP with *C*. *trachomatis* inclusions. NIH 3T3 fibroblasts which constitutively express individual fluorescent proteins, or those fused to the mGBP2 isoprenylation mutant C586S were infected with *C*. *trachomatis* (*C*.*t*.) and treated with IFNγ at 3 hpi. Cells were fixed at 14 hpi, stained for the chlamydia inclusion membrane marker CT868 together with DAPI. Cells were analysed by confocal microscopy and representative images are shown, bar = 2 μm.
(TIF)

S1 Video Dynamics of mGBP9 recruitment around the chlamydia inclusion. NIH 3T3 fibroblasts which stably express mCherry-fused mGBP9 were infected with *C*. *trachomatis* SW2::GFP and stimulated with IFNγ at 3 hpi. The cells were incubated at 37°C with 8% CO2 and humidity saturated air while recording confocal LSM images every 10 minutes. Video starts at 6h 40min p.i. bar = 5μm.
(MOV)
