# Supplementary figures and images for: Broad recruitment of mGBP family members to Chlamydia trachomatis inclusions
Source: PLoS One. 2017 Sep 25;12(9):e0185273. doi: 10.1371/journal.pone.0185273 (PMC5612764; doi:10.1371/journal.pone.0185273)

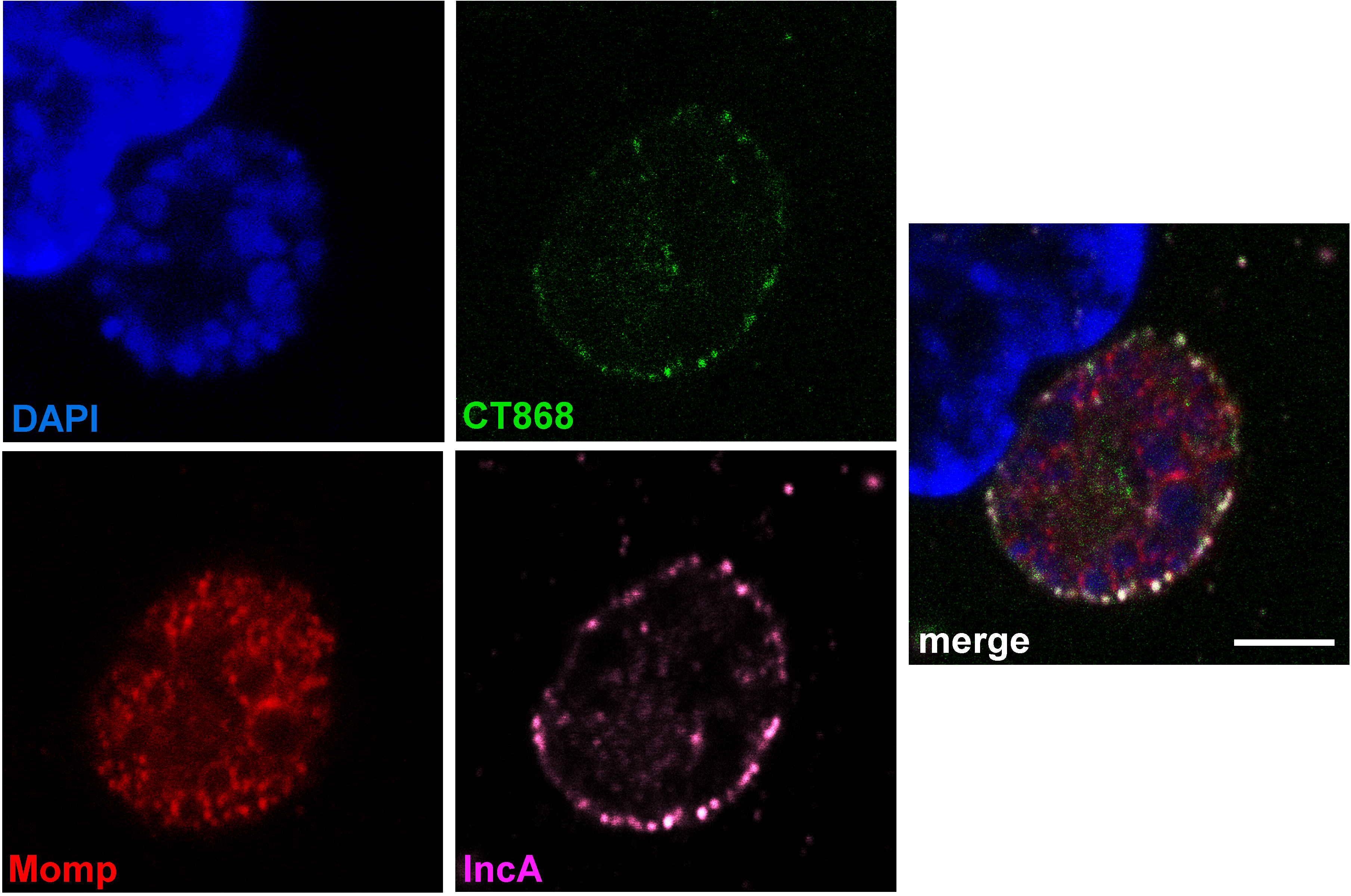

Supplement: S1 Fig — Staining: 24 hpi cells infected with C. trachomatis were fixed with 3% PFA, permeabilized with 0.5% Saponin and stained with antibodies against Momp and IncA in combination with anti-rabbit Alexa594 and anti-mouse Alexa647. Cells were fixed again in 3% PFA, permeabilized with 0.5% Saponin and CT868 was stained by anti-CT868 directly labeled with FITC. DNA was visualized by DAPI, bar = 5 μm. (TIF) [file pone.0185273.s001.tif]

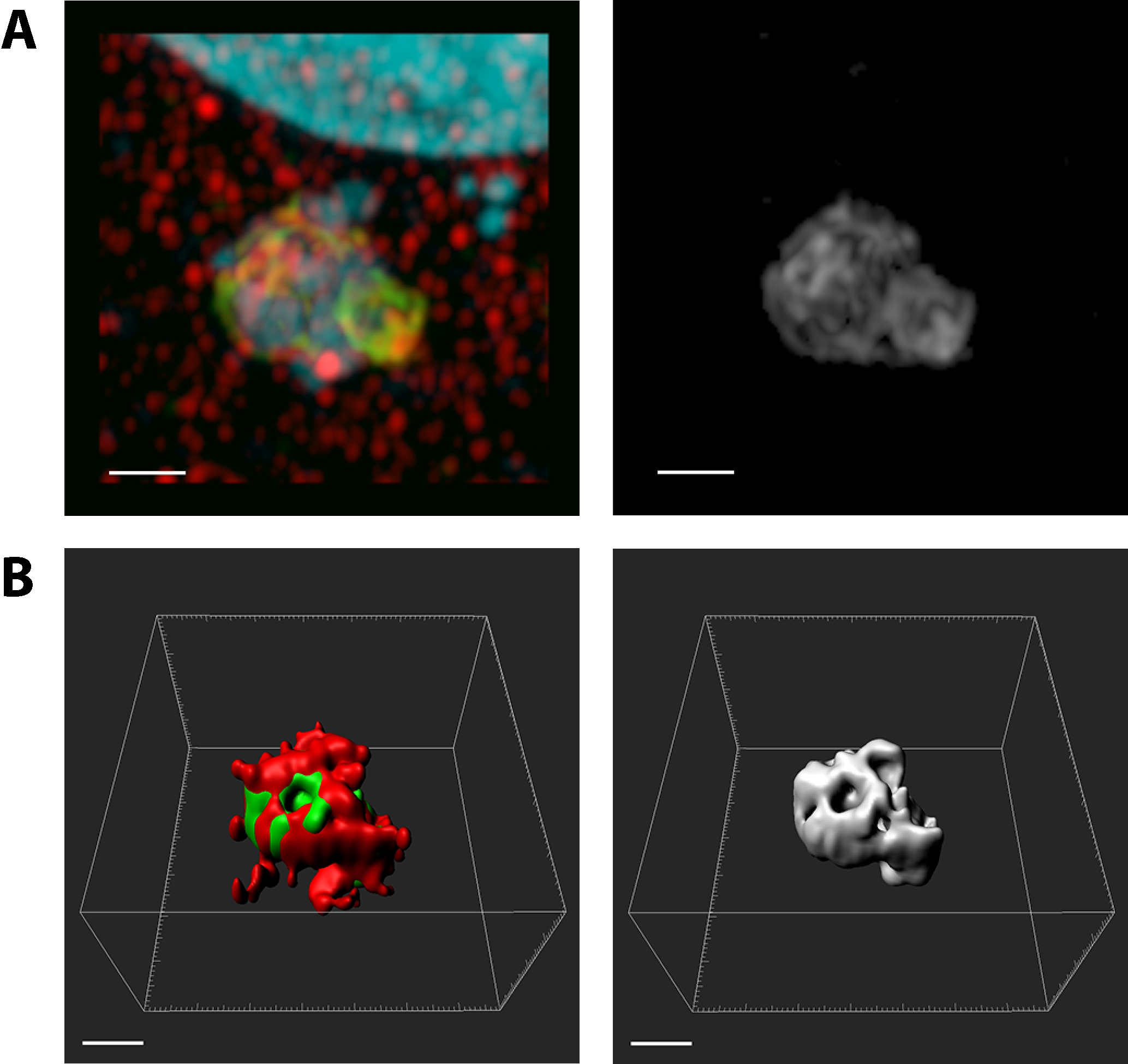

Supplement: S2 Fig — NIH 3T3 fibroblasts which stably express mCh::mGBP2 were infected with C. trachomatis and treated with IFNγ at 3 hpi. Cells were fixed at 14 hpi, stained for chlamydia inclusion membrane marker CT868 together with DAPI. Cells were analysed by confocal Airyscan microscopy and a z-stack was performed. A representative cell is shown. Colocalization and surface analysis was performed with Imaris. Bar = 1 μm. (A) left panel: maximum intensity projection of mCh::mGBP2 (red), CT868 (green) and DAPI (cyan). Right panel: computed colocalization of mCh::mGBP2 and CT868 is shown (grey). (B) surface analysis of the inclusion shown in A. Left panel: mCh::mGBP2(red) and CT868 (green). Right panel: computed colocalization of mCh::mGBP2 and CT868 (grey). (TIF) [file pone.0185273.s002.tif]

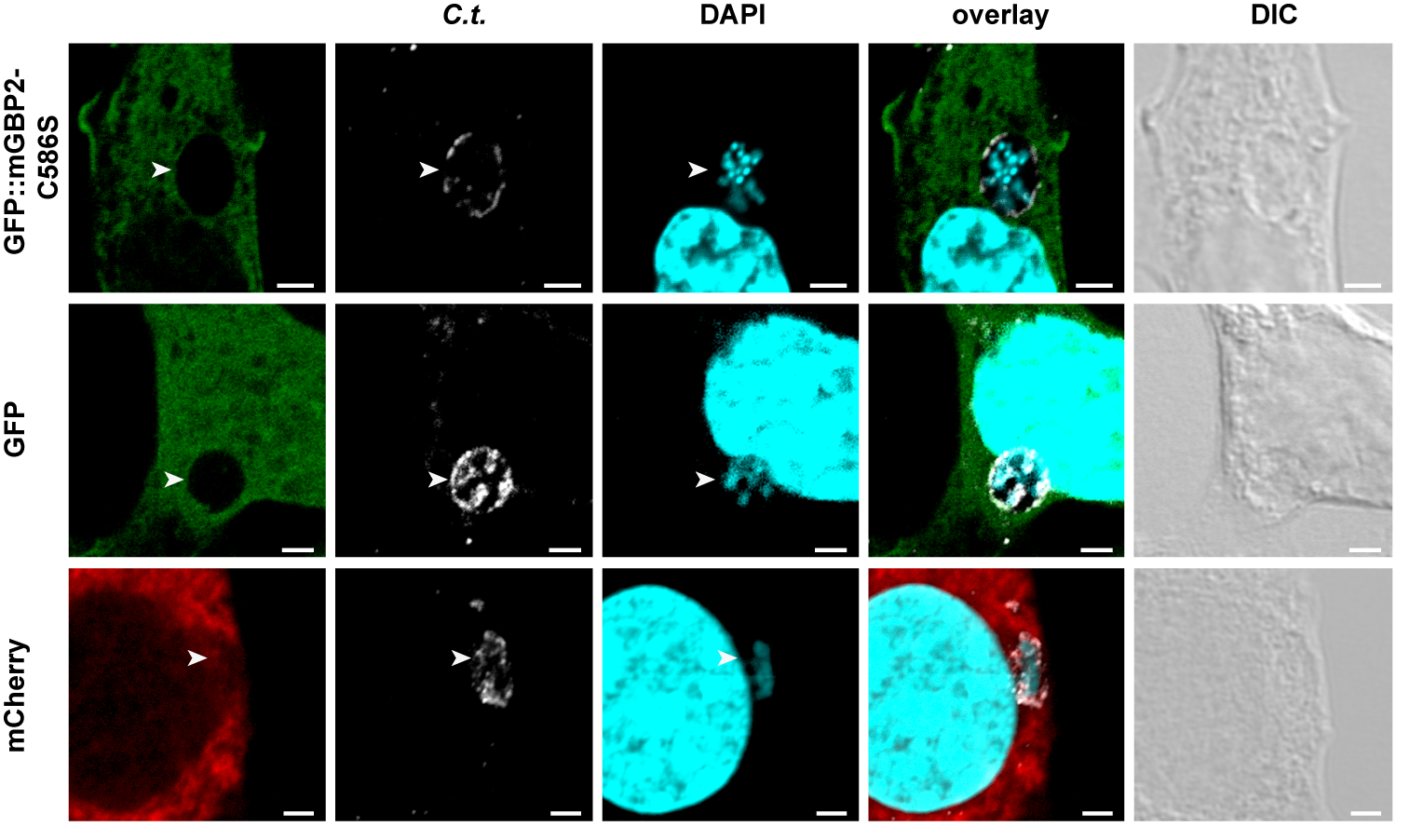

Supplement: S3 Fig — NIH 3T3 fibroblasts which constitutively express individual fluorescent proteins, or those fused to the mGBP2 isoprenylation mutant C586S were infected with C. trachomatis (C.t.) and treated with IFNγ at 3 hpi. Cells were fixed at 14 hpi, stained for the chlamydia inclusion membrane marker CT868 together with DAPI. Cells were analysed by confocal microscopy and representative images are shown, bar = 2 μm. (TIF) [file pone.0185273.s003.tif]
